# A secreted PD-L1 splice variant that covalently dimerizes and mediates immunosuppression

**DOI:** 10.1007/s00262-018-2282-1

**Published:** 2018-12-18

**Authors:** Kathleen M. Mahoney, Sachet A. Shukla, Nikolaos Patsoukis, Apoorvi Chaudhri, Edward P. Browne, Arnon Arazi, Thomas M. Eisenhaure, William F. Pendergraft, Ping Hua, Hung C. Pham, Xia Bu, Baogong Zhu, Nir Hacohen, Edward F. Fritsch, Vassiliki A. Boussiotis, Catherine J. Wu, Gordon J. Freeman

**Affiliations:** 1 Department of Medical Oncology, Dana-Farber Cancer Institute, Harvard Medical School, 450 Brookline Ave., Boston, MA 02215 USA; 2 Division of Hematology and Oncology, Beth Israel Deaconess Medical Center, Harvard Medical School, Boston, MA 02215 USA; 3 Department of Medicine, Massachusetts General Hospital Cancer Center, Boston, MA 02114 USA; 4 Broad Institute of the Massachusetts Institute of Technology (MIT) and Harvard, Cambridge, MA 02142 USA; 5 Department of Medicine, University of North Carolina Kidney Center, Chapel Hill, NC 27599 USA; 6 Neon Therapeutics Inc., Cambridge, MA 02139 USA

**Keywords:** PD-L1, Splice variants, Immune checkpoint, Isoforms

## Abstract

Targeting immune checkpoint pathways, such as programmed death ligand-1 (PD-L1, also known as CD274 or B7-H1) or its receptor programmed cell death-1 (PD-1) has shown improved survival for patients with numerous types of cancers, not limited to lung cancer, melanoma, renal cell carcinoma, and Hodgkin lymphoma. PD-L1 is a co-inhibitory molecule whose expression on the surface of tumor cells is associated with worse prognosis in many tumors. Here we describe a splice variant (secPD-L1) that does not splice into the transmembrane domain, but instead produces a secreted form of PD-L1 that has a unique 18 amino acid tail containing a cysteine that allows it to homodimerize and more effectively inhibit lymphocyte function than monomeric soluble PD-L1. We show that recombinant secPD-L1 can dimerize and inhibit T-cell proliferation and IFN-gamma production in vitro. The secPD-L1 variant is expressed by malignant cells in vitro that also express high levels of full-length PD-L1. Transcriptomic analysis of gene expression across The Cancer Genome Atlas found the strongest association of secPD-L1 with full-length PD-L1, but also with subsets of immunologic genes, such as in myeloid-derived suppressor cells. Moreover, the splice variant is also expressed in normal tissues and within normal peripheral blood cells it is preferentially expressed in activated myeloid cells. This is the first report of a form of secreted PD-L1 that homodimerizes and is functionally active. SecPD-L1 may function as a paracrine negative immune regulator within the tumor, since secPD-L1 does not require a cell-to-cell interaction to mediate its inhibitory effect.

**Electronic supplementary material:**

The online version of this article (10.1007/s00262-018-2282-1) contains supplementary material, which is available to authorized users.

## Introduction

Targeting immune checkpoints, such as programmed cell death-1 (PD-1) and its ligand programmed death ligand-1 (PD-L1, also known as CD274 or B7-H1) has revolutionized the treatment of patients with cancer. PD-L1 is a cell-surface ligand of the PD-1 and B7-1 receptors on lymphocytes. PD-L1 is expressed on immune cells, such as resting T cells, B cells, dendritic cells, and macrophage, as well as nonhematologic cells, such as the placenta. The interaction between the ligand PD-L1 and its receptor PD-1 mediates lymphocyte dysfunction, and blocking this interaction can restore lymphocyte function [3]. PD-L1 expression by tumor cells is associated with worse prognosis for patients with many types of tumor, such as kidney cancer [4]. Patients with higher levels of soluble PD-L1 detected in peripheral blood have worse clinical outcomes in both solid and hematologic malignancies, including kidney, lung, and hepatocellular carcinoma, as well as myeloma and diffuse large B cell lymphoma [5–9]. Whether soluble PD-L1 in patients is a surrogate for expression of PD-L1 by the tumor, be it tumor cells or the infiltrating immune cells, or a measure of immunosuppressive peripheral blood cells is not well established across tumor types. Whether the soluble PD-L1 can deliver a negative regulatory signal through PD-1 is debatable [10].

**Fig. 1:**
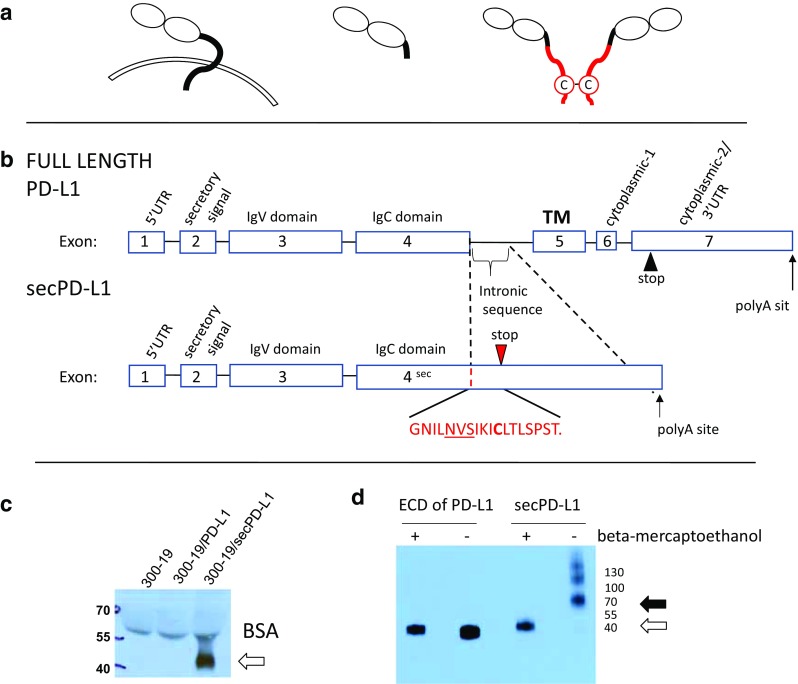
A splice variant of PD-L1 mRNA encodes a soluble form of PD-L1 that can covalently dimerize. **a** Protein schema of transmembrane PD-L1 (left) and a soluble PD-L1 cleaved from the cell surface (middle) and the dimeric secPD-L1 splice variant (right, C indicates a cysteine in the carboxyl-terminal domain). **b** Exons and introns of full-length PD-L1 (top) and secPD-L1 splice variant with unique cysteine-containing carboxyl-terminal domain (bottom; N-linked glycosylation motif (NVS) underlined). **c** Western blot analysis of PD-L1 in cell-free supernatants from 300.19 cells expressing either full-length PD-L1 or secPD-L1 (open arrow). **d** Western blot analysis of recombinant extracellular domain of PD-L1 and secPD-L1, in reducing and non-reducing conditions (open arrow indicating monomeric PD-L1 and closed arrow indicating dimeric secPD-L1 protein)

PD-L1 expression on tumor cells can be a result of viral activation [11], oncogene expression, genomic changes in the tumor, such as gene amplification or disruption of the 3′ untranslated region [12, 13]. PD-L1 expression can also be induced by IFN-γ, termed adaptive resistance [14]. PD-L1 is a transmembrane protein, which contains 7 coding exons, including a secretory signal at the amino terminus, IgV and IgC domains, a transmembrane domain, a short cytoplasmic tail, and a long 3′ untranslated region (Fig. 1a left, b upper). A soluble form of PD-L1 can be produced by tumor cell lines that express PD-L1 in vitro and by activated monocyte-derived dendritic cells that also express high levels of PD-L1 [6, 15]. It has been reported that soluble PD-L1 retains its immunosuppressive function [6] and can be generated by cleavage from the surface of cells by matrix metalloproteases (Fig. 1a, middle [16]). High levels of soluble PD-L1 in sera of patients with melanoma prior to immune checkpoint therapy were associated with increased likelihood of progressive disease after treatment with CTLA-4 blocking antibodies [17]. Regulation of PD-L1 at genetic, epigenetic, transcriptional, translational, and post-translational levels has been described [18, 19]. Few splice variants of PD-L1 have been described or functionally characterized. In adult T-cell leukemia/lymphoma, splice variants of PD-L1 have been described that affect the last 2 exons of the gene, encoding the cytoplasmic domain and 3′ untranslated region, enhancing mRNA stability and resulting in higher levels of expression of PD-L1 on the cell surface [13]. Multiple splice variants of PD-L1 have been reported that do not include the transmembrane domain but include more downstream exons, and thus may produce soluble forms of PD-L1 [17]. While it has been reported that molecularly engineered high-affinity PD-L1 can inhibit lymphocyte activation, B7 family proteins such as PD-L1 classically require multimeric binding to mediate signaling through their CD28 family receptors [20]. Thus native monomeric soluble PD-L1 would not be expected to functionally inhibit lymphocytes. Here we describe an alternative splice variant of PD-L1 that encodes a secreted form of PD-L1 (secPD-L1) with a unique cysteine-containing 18-amino acid domain which can dimerize. secPD-L1 can inhibit lymphocyte function in vitro. We show that cells expressing high levels of the full-length PD-L1 protein also express this secreted variant in vitro including tumors in the TCGA and some normal tissues. This secreted variant of PD-L1 may be an additional means by which cells can regulate T-cell function in the tumor microenvironment without requiring cell–cell interaction.

## Methods

*Isolation of secPD-L1 cDNA* Placenta RNA was purchased from Clontech Laboratories, Inc and was used to make the cDNA library [3]. A Rec-A-based system was used to clone PD-L1 cDNAs (Clone Capture kit) including full-length membrane and secreted isoforms by hybridization to plasmid cDNA libraries prepared from placenta mRNA [21, 22].

*Cell lines* All tumor cell lines were maintained as described previously [23].

*PCR analysis of RNA expression in cell lines and activated dendritic cells* RNA was isolated with RNAeasy Kit, reverse transcribed and PCR of full-length PD-L1 and the secreted variant of PD-L1 (secPD-L1) crossing exon–exon junctions was performed. To amplify the secPD-L1 mRNA qualitatively, PCR products after 30 cycles of amplification with O-3806 [crosses exon 3–4] (F1:ACTGTGAAAGTCAATGCCCC) and O-3816 [within the intron after exon 4] (R1: GCTAGGGGACAGTGTTAGAC, product 354 bp) or O-3818 [more 3′ within the intron after exon 4] (R2: GGATGAATGGAGGTGAGGAA, product 465 bp) were analyzed; under the same conditions we amplified the full-length PD-L1 mRNA with O-3808 [crosses exon 4–5 junction] (F2: ACAGCTGAATTGGTCATCCC) and O-3820 (R3: CTTGGAGGCTCCTTGTTCAG, product 505 bp) or O-3822 (R4: AGGGATTCTCAACCCGTCTT, product 550 bp) (Supplemental Fig. 2B upper, 2C). Quantitative PCR requires a shorter secPD-L1 PCR product for parallel PCR efficiency, which could also result in amplification of genomic DNA; thus RNA was treated with DNAse prior to cDNA production. TaqMan PD-L1 primers were used to detect mRNA expression of the transmembrane domain containing form of full-length PD-L1 or the unique 3′ sequence of secPD-L1, respectively: full-length PD-L1 (cat# Hs01125299_m1) and secPD-L1 (Cat# 4331348; ID: AI0IYL3) (schema in Supplemental Fig. 2B lower) and 18S control. The figure is representative of 3 or more Q-PCR experiments.

*Expression quantification of the PD-L1 isoforms* We first created a list of 36-mers tags derived from the secreted (secPD-L1) and the full-length membrane-bound (full-length PD-L1) transcriptomic isoforms of PD-L1. In a given RNA-seq library, reads deriving from either of these two isoforms were identified based on perfect matches to any tag in the list. Identified reads were then aligned to the secPD-L1 and full-length PD-L1 isoforms using a precise alignment method (Novoalign, http://www.novocraft.com), we defined the following quantities:

${n_{{\text{full-length~PD-L1}}}}$ is the # reads mapping to the 804 bases uniquely found at the 3′end of the full-length PD-L1 isoform; ${n_{{\text{secPD-L1}}}}$ is the # reads mapping to the 208 bases uniquely found at the 3′end of the secPD-L1 isoform; $N$ is the # reads in the RNA-seq library.

Normalized full-length and secPD-L1 counts were calculated as:

$$n_{{{\text{full-length}}\;{\text{PD-L1}}}}^{{{\text{norm}}}}=\frac{{{n_{{\text{full-length}}\;{\text{PD-L1}}}} \times {{10}^8}}}{N},$$

$$n_{{{\text{secPD-L}}1}}^{{{\text{norm}}}}=\frac{{{n_{{\text{secPD-L}}1}} \times {{10}^8}}}{N}.$$

The relative expression of full-length and secPD-L1 was calculated as:

$${\text{Rati}}{{\text{o}}_{{\text{full-length~:sec}}}}=\frac{{{n_{{\text{full-length}}\;{\text{PD-L1}}}}/804}}{{{n_{{\text{secPD-L1}}}}/208}}.$$

Samples with normalized isoform counts above 0 were considered positive for that isoform. Each sample was assigned to 1 of 4 classes: full-length+ sec+, full-length + sec-, full-length− sec+, full-length− sec− based on the status of the secPD-L1 and full-length PD-L1 isoforms within the sample. The gradient of color is used to denote the expression level of the full-length PD-L1 in any tumor expressing full-length PD-L1 or expression level of secPD-L1 in the secPD-L1 exclusive (yellow) tumor specimens.

*Transcriptomic databases* Expression levels of full-length PD-L1 and secPD-L1 were analyzed in publicly available tumor specimens from The Cancer Cell Line Encyclopedia (CCLE), The Cancer Genome Atlas (TCGA), normal tissue specimens prepared from autopsy [Genotype-Tissue Expression (GTEx) database], melanoma specimens from patients treated with ipilimumab or PD-1 therapy [24, 25]. The data used for the analyses described in this paper were obtained from the GTEx Portal dbGaP accession number phs000424.v6.p1. Sorted peripheral blood cells were sequenced per protocol from normal healthy donors [University of North Carolina Internal Review Board (IRB) # 13-3774].

*Recombinant secPD-L1* To confirm expression and secretion of the secPD-L1 protein, we subcloned the cDNA into the pEF-Puro expression plasmid and transfected 300.19 cells, a mouse pre-B cell line and assayed cell-free supernatants. Recombinant His-tagged secPD-L1 was produced in CHO cells and purified by its C-terminal His tag for functional assays and verified to have endotoxin levels below 2 EU/mg of protein. To assess multimerization of PD-L1, Western blot analysis of recombinant proteins following SDS–PAGE with and without beta-mercaptoethanol was performed on the PD-L1 extracellular domain (Met 1-Thr 239) with a C-terminal hexahistidine tag, which will be referred to as ECD-PD-L1 (Sino Biological 10084-H08H), and the purified recombinant secPD-L1. The PD-L1 monomer and multimers were detected with a murine monoclonal PD-L1 antibody that detects an epitope in the IgV domain of the extracellular domain of human PD-L1 (clone 368A.5A4, 0.25 µg/ml) [23]. In-fusion-mediated site-directed mutagenesis was used to mutate cysteine 239 to serine to determine the role of cysteine 239 in multimerization of secPD-L1 [26]. Expression of PD-L1 by cells transfected with vector, secPD-L1 or full-length PD-L1 was assayed by flow cytometry with anti-human PD-L1 (clone 339.6A2).

*Lymphocyte activation assays* PBMCs were purified by Ficoll gradient and T cells were isolated with Miltenyi beads (PanT cell kit, Cat# #130-096-535) and activated by soluble anti-CD3 and anti-CD28 antibodies (Fitzgerald Industries) at 100 ng/ml each in the presence of recombinant secPD-L1-His or the ECD-PD-L1 at increasing doses (5, 10, and 20 µg/ml). To assess the effect of secPD-L1 and ECD-PD-L1 on T-cell activation, supernatants were analyzed for IFN-γ production (Biolegend, Cat# 430102) at 48 h of stimulation. Proliferation was assessed by [^3^H]-thymidine incorporation for the last 16–18 h of 72-h cultures [27]. The figure is representative of two lymphocyte activation experiments.

## Results

*A secreted splice variant of PD-L1 is expressed by human placenta* PD-L1 is a glycosylated Type I membrane protein of the B7-family of immunomodulatory ligands. PD-L1 is highly expressed on some normal tissue such as the placenta, as well as on many hematologic cells. We cloned full-length (Fig. 1a, left; b, top) and alternatively spliced forms of the human PD-L1 cDNA from placental tissue by a hybridization method with RecA-coated biotinylated DNA probes [3, 21]. Sequencing of clones revealed an alternate splice variant, which we refer to as secPD-L1 (Fig. 1a, right, b, bottom), that contains the first 4 exons of PD-L1, including the secretory signal at the N terminus, IgV and IgC domains, which are shared with the full-length PD-L1. However, secPD-L1 does not splice into the fifth exon, which encodes the transmembrane domain, but reads into the fourth intron that results in a new stop codon, alternate 3′ untranslated region and polyadenylation site. Thus, the secPD-L1 splice variant produces an mRNA that contains a secretory signal at the 5′ end, lacks a transmembrane domain at its 3′ end and has 207 base pairs that differ from the 3′ end of the full-length PD-L1 mRNA (Supplemental Fig. 1a), and predicts a protein with a unique carboxyl terminal sequence: GNILNVSIKI**C**LTLSPST (Fig. 1b, bottom; Supplemental Fig. 1a).

*The secPD-L1 splice variant encodes a secreted protein and recombinant secPD-L1 dimerizes* We cloned the secPD-L1 cDNA into expression vectors and confirmed that this cDNA encodes a secreted protein by stably transfecting cells with the secreted splice variant of PD-L1 (secPD-L1), full-length PD-L1, or empty vector. Assaying supernatants from transfected cells by western blot analysis confirmed the secPD-L1 protein was secreted with an estimated size of 42 KD (Fig. 1c). Flow cytometry of transfected cells showed little PD-L1 was expressed on cells transfected with secPD-L1 compared to cells transfected with full-length PD-L1 (Supplemental 1b). The secPD-L1 unique C-terminal domain contains a cysteine, which suggests that secPD-L1 may homodimerize (Fig. 1b, bottom). Using a PD-L1 antibody that recognizes an epitope in the IgV domain, we performed western blot analysis of recombinant secPD-L1 and compared it to recombinant extracellular domain of PD-L1 under non-reducing and reducing conditions to determine whether the secPD-L1 variant migrated differently than a form of PD-L1 cleaved from the full-length membrane protein (Fig. 1d). In reducing conditions, both migrated at about 40 KD; however, in non-reducing conditions, the recombinant extracellular domain of PD-L1 remained monomeric, while the secPD-L1 had a molecular weight consistent with a dimeric structure with some larger multimers. Mutation of cysteine 239 to serine in the recombinant secPD-L1 largely abrogated multimerization of the isoform (Supplemental Fig. 1c).

**Fig. 2:**
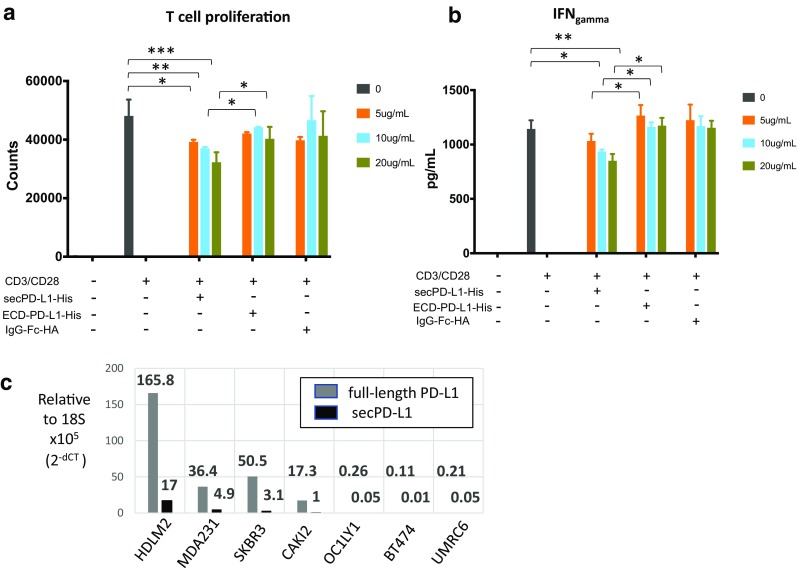
SecPD-L1 is functional and is expressed in PD-L1 “positive” tumor cells. **a** Proliferation of CD3/CD28 activated T lymphocytes in the presence of recombinant secPD-L1, the soluble monomeric extracellular domain of PD-L1 (ECD-PD-L1) or an immunoglobulin-HA tagged fusion protein as control after 72 h (*p value < 0.05, **p value < 0.005, ***p value < 0.0005). **b** IFN-γ production of CD3/CD28-activated T lymphocytes after treatment as in **a** was assayed after 48 h. **c** Quantitative RT-PCR of full-length and secPD-L1 in tumor cell lines (lymphoma: HDLM2, OC1-LY1; kidney cancer: CAKI-2, UMRC6; and breast cancer: SKBR3, MDA231 and BT474)

*SecPD-L1 is immunosuppressive in vitro* Soluble PD-L1 has been reported to bind PD-1 and retain immunosuppressive activity in vitro. If signaling is dependent on crosslinking the PD-1 receptor, a monomeric cleaved form of PD-L1 would not be expected to be functional. Functional assays performed with recombinant PD-L1-Fc fusion proteins allow for dimerization by the Fc portion of the recombinant protein [6]. Given that the secPD-L1 variant can naturally dimerize, we compared the functional activity of recombinant secPD-L1 and a recombinant PD-L1 containing only the extracellular domain without the sec-specific carboxyl terminus and similarly purified with a His tag, rather than an Fc fusion, to determine whether secPD-L1 has immunosuppressive activity. We found that secPD-L1 at 10 µg/ml concentrations could inhibit T-cell proliferation and production of IFN-γ from T cells stimulated with CD3/CD28 coactivation, and was more inhibitory than the soluble extracellular domain of PD-L1 (Fig. 2a, b).

*SecPD-L1 RNA is expressed in PD-L1-positive malignant cell lines* We have developed monoclonal antibodies (mAb) to detect PD-L1 in flow cytometry, western blot, and immunohistochemical analysis [22, 23, 28]. Within this set of tools, we have mAbs that recognize distinct domains within PD-L1 (IgV, IgC, cytoplasmic). While we have developed an ELISA assay to detect soluble PD-L1 [17], our attempts to produce high-affinity antibodies specific for the unique 18 amino acids at the carboxyl terminus of secPD-L1 to distinguish whether soluble PD-L1 in patient sera is the secreted variant described here have not yet been successful. A challenge in developing an antibody specific for the secPD-L1 variant may be due to the C-terminal region of secPD-L1 having an N-linked glycosylation motif (NVS, underlined in Fig. 1b) that could block mAb access. However, since there is a distinct 3′ nucleotide sequence in the carboxyl terminus and 3′UTR that differ between the full-length and secPD-L1, we were able to measure mRNA expression of the secPD-L1 variant in a series of cell lines by RT-PCR. We have previously reported immunohistochemistry and western blot analysis with the 405.9A11 mAb, which recognizes an epitope in the cytoplasmic domain of full-length PD-L1, and corresponded with surface expression of PD-L1 by flow cytometry and a membranous pattern of expression of PD-L1 on tumor cells [23]. We screened a series of cell lines with established positive or negative PD-L1 protein status by flow cytometry and western blot analysis, including lymphomas [HDLM-2(+) and OC1-LY1(−)] [12], renal cell carcinoma [Caki-2(+), UMRC6(−)] [23], and breast cancer cell lines [MDA231(+), SKBR3(+), and BT474(-)] [28] to determine whether the secPD-L1 variant mRNA expression was associated with expression of the full-length PD-L1 by RT-PCR analysis. PCR with exon–exon bridging primers established that cell lines expressing the full-length PD-L1 (HDLM2, Caki-2, MDA231, and SKBR3) also expressed secPD-L1 mRNA (Supplemental Fig. 1b, c). The exon–exon bridging PCR product for the secPD-L1-specific product was > 400 bp given the shared exon 4 sequence between the full-length and secPD-L1 cDNA (Supplemental Fig. 1b, c). To compare relative amounts of full-length and secPD-L1 by quantitative real-time PCR, we used a standard set of TaqMan primers specific for the cDNA encoding the transmembrane domain or custom TaqMan primers specific for the unique intronic sequence encoded in secPD-L1 on DNAse-treated RNA samples (Fig. 2c). In our panel of cell lines, HDLM2, a Hodgkin lymphoma line, expressed the highest levels of full-length PD-L1 and secPD-L1 RNA compared to other PD-L1-expressing cell lines, SKBR3, MDA231, and Caki-2. This was expected since Hodgkin lymphomas express high levels of full-length PD-L1 protein often due to a genomic amplification of the 9p24 region that encodes PD-L1 gene [12]. Cell lines with undetectable levels of full-length PD-L1 by Western and flow cytometry also expressed extremely low levels of PD-L1 mRNA and secPD-L1 mRNA by quantitative RT-PCR. Of note, full-length PD-L1 was expressed at 5–16fold higher levels than secPD-L1 within each cell line expressing full-length PD-L1 tested in the representative experiment in Fig. 2c.

**Fig. 3:**
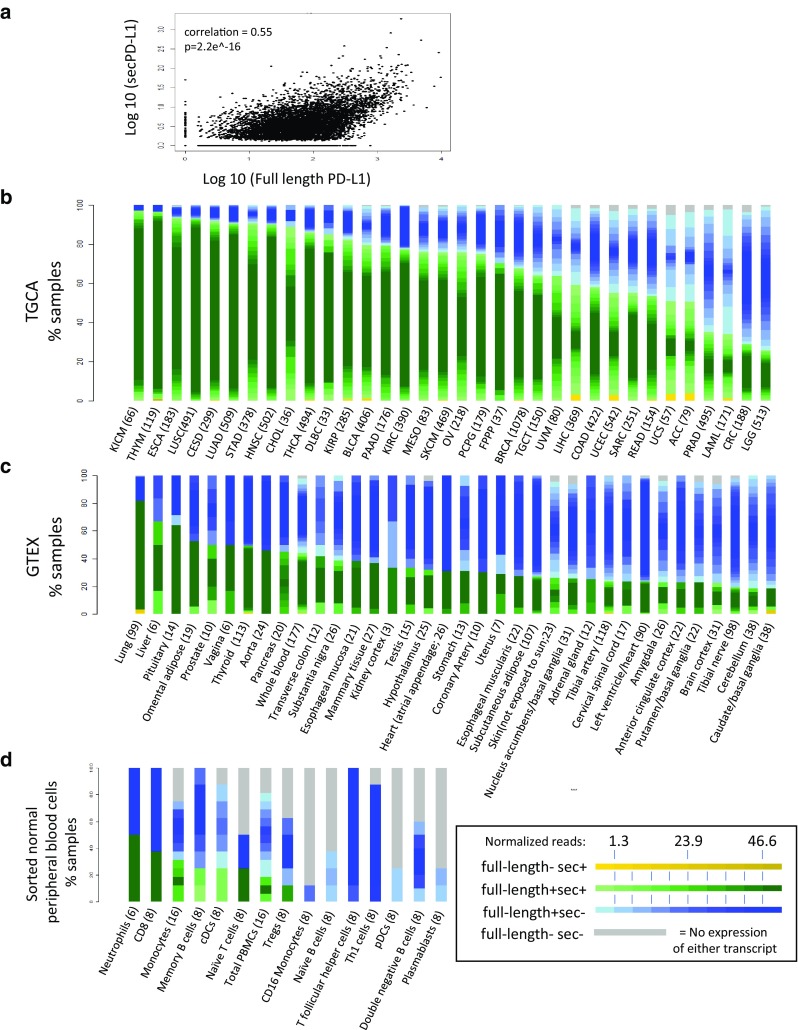
SecPD-L1 RNA expression is associated with full-length PD-L1 expression and is found in the majority of human tumors and also some normal hematologic cells. **a** Correlation of secPD-L1 expression with full-length PD-L1 RNA expression in The Cancer Genome Atlas (TCGA) data (normalized to total RNA transcripts, *n* = 9677 tumors, correlation = 0.55, *p* = 2.2e^− 16^). **b** Frequency of secPD-L1 and/or full-length PD-L1 within each tumor type in the TCGA. Each specimen was assigned to one of four classes: full-length− sec+ (yellow shades), full-length + sec+ (green shades), full-length+ sec− (blue shades), full-length− sec− (grey) based on the frequency of the secPD-L1 and full-length PD-L1 isoforms (number of specimens per tumor type listed in parenthesis). The expression value of a sample within any class was color-shaded as follows. The interquartile range of the overall distribution of both isoforms was divided into 10 equal groups, with the first and last groups subsuming values ≤ 25th and ≥ 75th quartile, respectively. The expression of a sample within a class was represented by the appropriate color on the class-specific color gradient (color gradient created using colorRampPalette in R; full-length− sec+: yellow–yellow, full-length + sec+: light green–dark green, full-length+ sec−: light blue–blue, full-length− sec−: grey). The level of full-length PD-L1 counts is represented by the gradient of color of the full-length+ sec+ and full-length+ sec− groups; the level of secPD-L1 count is represented by the color yellow gradient in the full-length− sec+ group. **c** Frequency of secPD-L1 and/or full-length PD-L1 in normal tissue in GTEx (number of specimens per tissue type in parenthesis). The expression of the full-length+ sec+ and full-length+ sec− classes was represented by the normalized full-length PD-L1 count while the full-length− sec+ group was represented by the corresponding normalized secPD-L1 count, with darker color representing higher expression of full-length PD-L1 or secPD-L1, respectively. **d** Some lymphocytes, myeloid cells and neutrophils expressing full-length PD-L1 also express secPD-L1 (secPD-L1+ and full-length PD-L1+ in green, secPD-L1 negative and full-length PD-L1+ in blue, and secPD-L1 negative and full-length PD-L1 negative in grey; transcripts normalized to total transcripts)

*Transcriptomic analysis of the TCGA database found that secPD-L1 frequently is expressed in a subset of patients across tumor types* The difference between the 3′ ends of the full-length and secPD-L1 allows for computational analysis of mRNA expression levels of full-length and secPD-L1 in normal and tumor databases. We first verified our methods of analysis (Supplemental Fig. 2a) by screening tumor cell line transcriptomes in the Cancer Cell Line Encyclopedia (CCLE) that we had established as PD-L1-positive by protein analysis, such as HDLM2, SKBR3, MDA231, and Caki-2 or negative (such as BT474). Interrogation of TCGA showed secPD-L1 expression correlated with full-length PD-L1 expression (corr = 0.55, *p* < 2.2e-16, Fig. 3a). 70% of tumors in TCGA (6871/9677) expressed some amount of secPD-L1 compared to 98.6% that expressed some amount of full-length PD-L1 (Table 1). We found that the full-length PD-L1 was expressed at higher amounts and in more tumors than secPD-L1 transcripts across tumor types, a pattern similar to that shown by qRT-PCR in cell lines (Fig. 2c). We divided each tumor type within TCGA into four categories based on whether they expressed secPD-L1 without full-length PD-L1 (yellow), full-length PD-L1 without secPD-L1 (blue), both full-length PD-L1 and secPD-L1 (green), or neither form of PD-L1 (grey) (Fig. 3b). The majority (median 75.5**%**, range 25.7–97.0) of tumor types in TCGA express both splice forms (Fig. 3b, green). Only 0.9% of total tumors in TCGA expressed secPD-L1 without full-length PD-L1 (Fig. 3b, yellow; Table 1). However, some tumor types had a small percentage that expressed only secPD-L1 (yellow, median 0%, range 0–3.8) with over 2% in liver hepatocellular carcinoma (LIHC), uterine corpus endometrial carcinoma (UCEC), uterine endometrial carcinoma (USC), and adrenocortical carcinoma (ACC), and acute myeloid leukemia (LAML). When we analyzed data from two small cohorts of previously reported patients with melanoma treated with immune checkpoint therapy [24, 25], we found nearly all melanoma specimens (98.5%) in both immune check point inhibitor-treated cohorts expressed some full-length PD-L1 (ipilimumab *n* = 40, PD-1 blocker *n* = 28), which was comparable to that (98.9%) in the cohort of cutaneous melanoma specimens in the TCGA (Supplemental Fig. 2d–e). In the cohorts of patients treated with a CTLA-4 or PD-1 blocker the majority of melanomas did express secPD-L1 (60 and 82%) and there was no association between clinical outcome and secPD-L1 mRNA expression in the tumor.

**Table 1:**
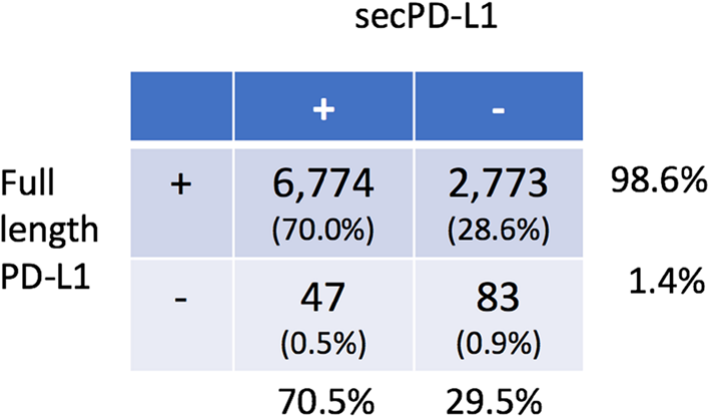
Full-length and secPD-L1 expression in tumors within TCGA

*Transcriptomic analysis of the GTEx database found that secPD-L1 can be expressed in normal tissue* To assess expression of secPD-L1 and full-length PD-L1 in normal tissues, we analyzed the GTEx database of RNASeq from specimens isolated from different human tissues (Fig. 2d). As seen in TCGA, the vast majority of normal tissue expressed some amount of full-length PD-L1 (green and blue). However, there were fewer normal tissue specimens with expression of secPD-L1 (green and yellow). Of note, the expression of secPD-L1 was highest in the lung, liver, and pituitary, but the minority of normal tissues express detectable levels of secPD-L1 by this analysis. Thus, while secPD-L1 is modestly expressed in many normal tissues, secPD-L1 expression appears to be increased in tumors when comparing TCGA with GTEx (Fig. 3b, c, green and yellow bars, median 75.6 and 27.3%).

*secPD-L1 can be expressed by various hematologic cells, but is strongly associated with myeloid-derived suppressor cell-signatures in TCGA* While the full-length PD-L1 protein is expressed in some normal tissues, such as placenta and the eye, its most established role is in hematologic cells, such as dendritic cells, macrophages, and activated lymphocytes. Since TCGA and GTEx databases are produced from bulk RNASeq of tissue specimens, they do not distinguish whether the cell type expressing the transcript is tumor cell, epithelial cell, stromal or immune cells. Others have shown that activated monocyte-derived dendritic cells produce soluble PD-L1 by cleavage from the membrane [15] and the RNASeq analysis indicates that a fraction of monocytes and classical dendritic cells express secPD-L1. Therefore, we confirmed that activated monocyte-derived dendritic cells express secPD-L1 mRNA, as well as full-length PD-L1 mRNA by qRT-PCR (data not shown). To determine which distinct hematologic cell types from healthy donors expressed secPD-L1 and full-length PD-L1, we analyzed the RNA expression of sorted peripheral blood cells (Fig. 3d). We found that neutrophils, as well as some subtypes of lymphoid and myeloid cells express secPD-L1 RNA.

**Fig. 4:**
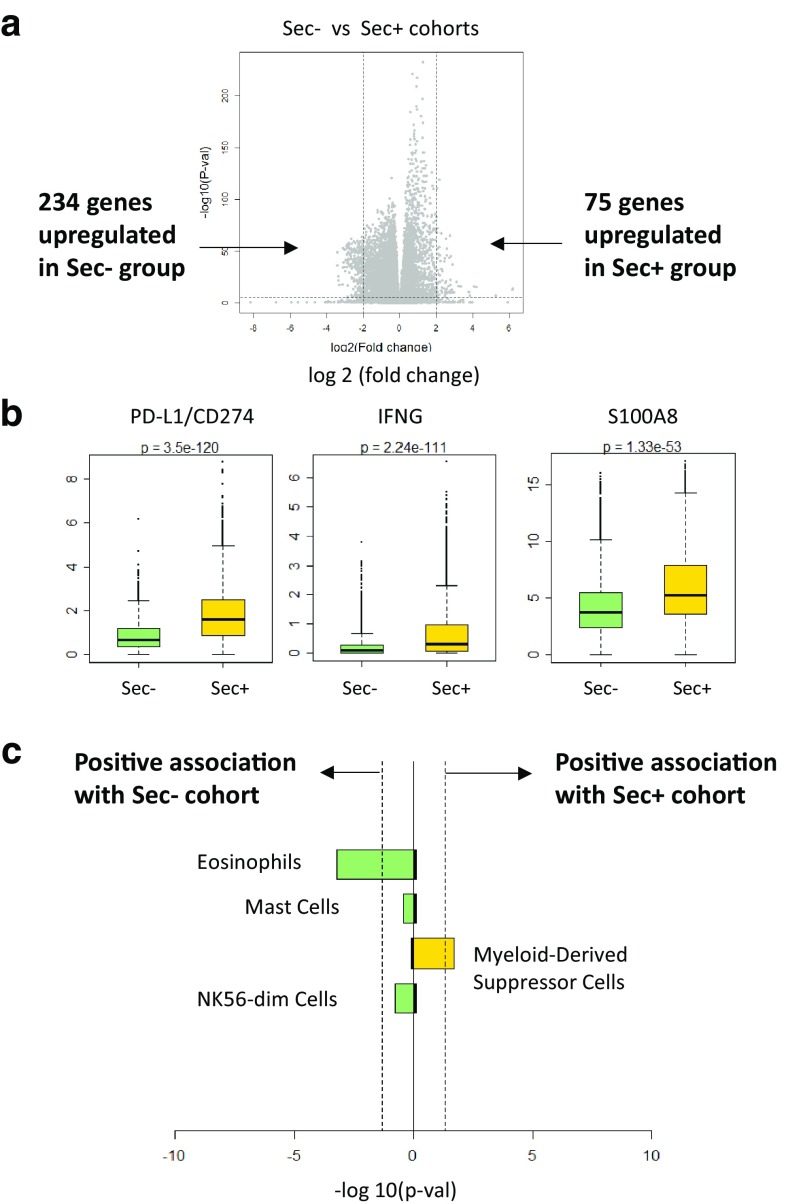
Gene Set Enrichment Analysis (GSEA) shows differences in sec-expressing and no-sec-expressing cohorts of TCGA. **a** Differential gene expression of the sec-expressing cohort (75 genes, FDR 5%, greater than twofold change in expression) and no-sec-expressing cohort (234 genes). **b** Within the 805 immune related genes in the differential gene analysis, PD-L1(CD274), IFNg, and S100A8 were the three most significantly enriched in sec-expressing cohort. **c** Zlatko’s gene sets overlap analysis indicates that the MDSC gene set is associated with sec + cohort (yellow)

Given that full-length PD-L1 is expressed in 98.6% of specimens in TCGA, while secPD-L1 is expressed in 70.5% of specimens, we performed differential expression analysis depending on whether tumors expressed any secPD-L1. Differential expression analysis of samples with detectable secPD-L1 (*n* = 6821) or no-secPD-L1 (*n* = 2856) found 75 genes and 234 genes with twofold or more increase in the secPD-L1 expressing and no-secPD-L1 cohorts, respectively (Fig. 4a, FDR 5%). Full-length PD-L1 (CD274) was the most significantly associated gene with the secPD-L1 expressing cohort, followed by IFN-γ and S100A8 (Fig. 4b). Compared to the no-secPD-L1 cohort, the secPD-L1 cohort expressed significantly higher levels of certain cytokines and chemokines, including IL21, CSF2, CCL25, and CCR9 (Supplemental Fig. 3a). Interestingly, Zlatko’s gene sets overlap analysis of sec-expressing and no-secPD-L1 expressing TCGA cohorts showed that the myeloid-derived suppressor cell gene set was associated with the sec-expressing cohort, while the eosinophil gene set was associated with no-secPD-L1 expressing cohort (Fig. 4c).

## Discussion

Tumor expression of PD-L1 inhibits the anti-tumor immune response and lets tumors evade immune attack [29, 30]. Targeting PD-1 and its ligand PD-L1 has shown outstanding clinical benefit in clinical trials, and has significantly less toxicity than IL-2 or CTLA-4 blockade, leading to their FDA approval for numerous indications, including not only melanoma and non-small cell lung cancer, but also kidney and bladder cancer, as well as others including refractory Hodgkin’s lymphoma. For lung cancer patients with high PD-L1 expression on tumor cells (> 50%), starting treatment with the PD-1 blocking therapy pembrolizumab results in better survival than starting with chemotherapy [31]. However the role of PD-L1 as a biomarker has not proven to be sufficiently definitive to be clinically useful in most other settings. In tumors such as kidney cancer, PD-L1 expression on the tumor cells has been correlated with tumor aggressiveness and shorter patient survival, but this did not translate into longer survival in “high” PD-L1-expressing kidney tumors when treatment with PD-1 blockade (nivolumab) was compared to everolimus in patients who had previously been treated with a VEGF tyrosine kinase inhibitor [4, 32]. While it is critical to determine what biomarkers are useful for predicting who will respond to PD-1 blockade alone and who will require combination therapy, surface expression of PD-L1 has not proven to be a simple or straightforward biomarker in most tumors at the protein or RNA level. A soluble form of PD-L1 also has been described and may be produced by multiple mechanisms, including cleavage from the surface of the cell [16] or alternate splicing [17]. This study is the first to assess the function of a secreted splice variant of PD-L1 that can dimerize and its expression in tumor and normal cells.

PD-L1 protein expression has been described both on tumor cells and on immune cells within the tumor microenvironment. Thus, stratifying PD-L1 expression of the tumor cell by RNA expression in bulk analysis of tumors is not feasible. For example in Hodgkin’s lymphoma, while PD-L1 is highly expressed on Hodgkin Reed-Sternberg cells due to a chromosomal amplification of the PD-L1 gene, the bulk of the tumor mass is normal immune cells [12]. Furthermore different cell-surface PD-L1 mRNA variants can produce higher levels of protein expression on the tumor cells’ surface, as shown by the effect of structural variations in the 3′ UTR region of PD-L1 mRNA found in a small subset of tumors [13]. In addition, expression of membrane-bound PD-L1 is regulated during the cell cycle by polyubiquitination of a lysine in the PD-L1 cytoplasmic domain; however, secPD-L1 lacks the cytoplasmic domain and therefore should be regulated in a different fashion. Most solid tumors’ PD-L1 expression is extremely variable and likely not driven by a single genetic driver mutation. Often the biomarker threshold of “positive” expression is relatively low at 1 or 10% tumor cells.

Little is known about the normal biology of splice variants of PD-L1. However in many studies, membranous expression of PD-L1 on tumor cells is used to determine specific PD-L1 expression. Whether some of the non-specific cytoplasmic staining seen with some antibodies is due to splice variants without the transmembrane domain has not been determined. The calculated size of unmodified secPD-L1 would be 26 KD but the observed 42 KD size in Fig. 1c is likely due to post-translational modifications such as glycosylation. Not only does PD-L1 have four glycosylation sites in the extracellular domain shared by secPD-L1 and full-length PD-L1, but the unique carboxyl terminus of secPD-L1 also contains an N-linked glycosylation site. Our analysis of TCGA shows that mRNA expression of full-length PD-L1 with a transmembrane domain was a log-fold greater than secPD-L1 expression (Supplemental 1C), suggesting full-length PD-L1 is the major form of PD-L1. In Genbank and as illustrated in Fig. 1, the full-length PD-L1 is variant 1, the longest form (isoform a). Variant 2 (isoform b) is a shorter protein that lacks the IgV exon necessary for binding the PD-1 receptor. Variant 3 contains multiple in-frame stop codons which likely results in a non-coding RNA. The secPD-L1 mRNA is transcript variant 4 (NCBI Reference Sequence: NM_001314029.1). Recently we reported additional splice variants of PD-L1 in melanoma tumors, which have the PD-1 binding domain and cytoplasmic exons but do not include the transmembrane domain [17]. The most significant difference in the secPD-L1 variant from previously described splice variants is the carboxyl terminus of secPD-L1 and its capacity to dimerize. Despite analyzing multiple thresholds of positive secPD-L1 and full-length PD-L1 in the clinical cohorts that we had access to, we did not find that secPD-L1 expression predicted clinical outcomes better than full-length PD-L1 expression. Therefore, in this study, we used a binary ± expression of secPD-L1 to present our transcriptomic analysis of secPD-L1 and full-length PD-L1. Others have shown the 3′ UTR regions of full-length PD-L1 mRNA contains regulatory regions that control mRNA stability. The unique 3′ UTR of secPD-L1 is different and contains fewer AUUUA motifs than full-length PD-L1 mRNA and consequently may be a more stable transcript than the full-length PD-L1 mRNA. More extensive proteomic analysis of large cohorts may determine whether secPD-L1 protein is a better biomarker than full-length PD-L1 in the right context.

Whether soluble PD-L1 in the peripheral blood of cancer patients is a useful biomarker is not yet clear. Circulating levels of soluble PD-L1 have been correlated with worse clinical features and outcomes across tumor types, including RCC, multiple myeloma, and diffuse large B-cell lymphoma [5–9]. In melanoma, high serum levels of PD-L1 prior to treatment with immune checkpoint therapy also are associated with progressive disease [17]. However, soluble PD-L1 in peripheral blood is not associated with worse prognosis in patients with pancreatic cancer; nor are soluble PD-L1 levels associated with PD-L1 expression on pancreatic tumor cells; but soluble PD-L1 levels are associated with markers of inflammation, such as C-reactive protein (CRP) and strong infiltration of T cells into the tumor [33]. Here, we show that secPD-L1 can be expressed by tumor and normal cells. The composition of the pool of soluble PD-L1 found in the peripheral blood likely includes secPD-L1 and a mixture of different variants of soluble PD-L1 cleaved from the surface and secreted alternative splice variants. Moreover, levels of soluble PD-L1 are elevated in pregnancy [34], and also in patients with pancreatitis associated with infectious complication [35], suggesting that elevated soluble PD-L1 levels may be immunosuppressive at a distance even in non-malignant settings.

Here we describe a secreted splice variant of PD-L1 (secPD-L1) which is expressed in the majority of cancers, can dimerize and inhibit activation of T lymphocytes in vitro. This secreted splice variant of PD-L1 was independently identified in a head and neck squamous cell carcinoma with a human papilloma virus integration in the PDL1 locus upstream of the transmembrane domain-encoding region, as described by Hassounah et al. in “Identification and characterization of an alternative cancer-derived PD-L1 splice variant”, which is being copublished with our work. The integration of human papilloma virus upstream of the transmembrane domain-encoding region favors the expression of the secreted form of PD-L1 and their analysis substantiates the expression of this secreted isoform of PD-L1 across numerous cancers. Furthermore, we show that this isoform is expressed by PD-L1-positive tumor cells, and also PD-L1-positive normal tissue, including myeloid cells, such as dendritic cells. Since secPD-L1 is not exclusive to the tumor cells, it can be inferred that soluble PD-L1 in the peripheral blood may not be a simple surrogate for PD-L1 expression by tumor cells. Moreover, the level of soluble PD-L1 is dynamic. After starting immune checkpoint therapy, levels of soluble PD-L1 often rise, though within the first few months of treatment, this change is not associated with better outcomes [17]. Yet patients who survive beyond 5 months and have higher serum PD-L1 levels were more likely to develop partial responses. We currently do not have a means to distinguish different protein forms of soluble PD-L1. Soluble PD-L1 found in serum may be produced by multiple cell types and through different mechanisms, such as cleavage from the cell surface by a matrix metalloprotease [16] or alternative expression of PD-L1 splice variants. Our data indicate that a monomeric form of soluble PD-L1 is less effective at inhibiting T-cell activation than secPD-L1. The biological activity of secPD-L1 required relatively high concentrations in vitro, suggesting that secPD-L1 might be most active in the tumor microenvironment rather than systemically, since the concentration of soluble PD-L1 in the peripheral blood of patients rarely reaches 10 µg/ml [5–9]. Since its inhibitory effect does not depend on a cell-to-cell interaction, it may be a novel mechanism of mediating immunosupression within the tumor microenvironment in a paracrine manner. The differences in immunologic gene expression in the sec-expressor cohort and no-secPD-L1-expresser cohort in TCGA illustrate that multiple immunologic pathways are active in the secPD-L1 expressing tumors. Further understanding the tumor biology of PD-L1 and its splice variants will improve our means of overcoming resistance to PD-L1/PD-1 pathway therapy.

## Electronic supplementary material

Below is the link to the electronic supplementary material.

Supplementary material 1 (PDF 2008 KB)

## Abbreviations

ACC: Adrenocortical carcinoma
ATCC: American Type Culture Collection
B7-1: First of the B7-family of immunomodulatory proteins
CCLE: The Cancer Cell Line Encyclopedia
CHO: Chinese hamster ovarian cells
CRP: C-reactive protein
CTLA-4: Cytotoxic T-Lymphocyte-Associated Protein 4
ECD-PD-L1: Extracellular domain of PD-L1 (Met 1-Thr 239)
GTEx: Genotype-Tissue Expression database
IFN-γ: Interferon-gamma
IgC domain: Protein domain structurally similar to the constant region of immunoglobulins
IgV domain: Protein domain structurally similar to the variable region of immunoglobulins
IRB: Internal Review Board
LAML: Acute myeloid leukemia
LIHC: Liver hepatocellular carcinoma
mRNA: Messenger RNA
NVS: N-linked glycosylation motif
PD-L1: Programmed death ligand-1
PD-1: Programmed cell death-1
RCC: Renal cell carcinoma
secPD-L1: Secreted splice variant of PD-L1
TCGA: The Cancer Genome Atlas
UCEC: Uterine corpus endometrial carcinoma
USC: Uterine endometrial carcinoma
VEGF: Vascular endothelial growth factor
