# Supplementary material for: A secreted PD-L1 splice variant that covalently dimerizes and mediates immunosuppression
Source: Cancer Immunol Immunother. 2018 Dec 18;68(3):421–32. doi: 10.1007/s00262-018-2282-1 (PMC6426808; doi:10.1007/s00262-018-2282-1)
Supplement: Supplementary file 1 — Supplementary material 1 (PDF 2008 KB) [file 262_2018_2282_MOESM1_ESM.pdf]

Supplemental Figure 1.

a. secPD-L1 amino acid sequence

MRIFAVFIFMTYWHLLNAFTVTVPKDLYVVEYGSNMTIECKFPVEKQLDLAALIVYWEMEDKNIIQFVHGEEDLKVQHSSYRQ
RARLLKDQLSLGNAALQITDVKLQDAGVYRCMISYGGADYKRITVKVNAPYNKINQRILVVDPVTSEHELTCQAEGYPKAEVIW
TSSDHQVLSGKTTTTNSKREEKLFNVTSTLRINTTTNEIFYCTFRRLDPEENHTAELVIP**GNILNVSIKICLTLSPST**.

Full length PD-L1 amino acid sequence

MRIFAVFIFMTYWHLLNAFTVTVPKDLYVVEYGSNMTIECKFPVEKQLDLAALIVYWEMEDKNIIQFVHGEEDLKVQHSSYRQ
RARLLKDQLSLGNAALQITDVKLQDAGVYRCMISYGGADYKRITVKVNAPYNKINQRILVVDPVTSEHELTCQAEGYPKAEVIW
TSSDHQVLSGKTTTTNSKREEKLFNVTSTLRINTTTNEIFYCTFRRLDPEENHTAELVIP**ELPLAHPPNERTHLVILGAILLCLGVAL
TFIFRLRKGRMMDVKKCGIQDTNSKKQSDTHLEET**.

b.

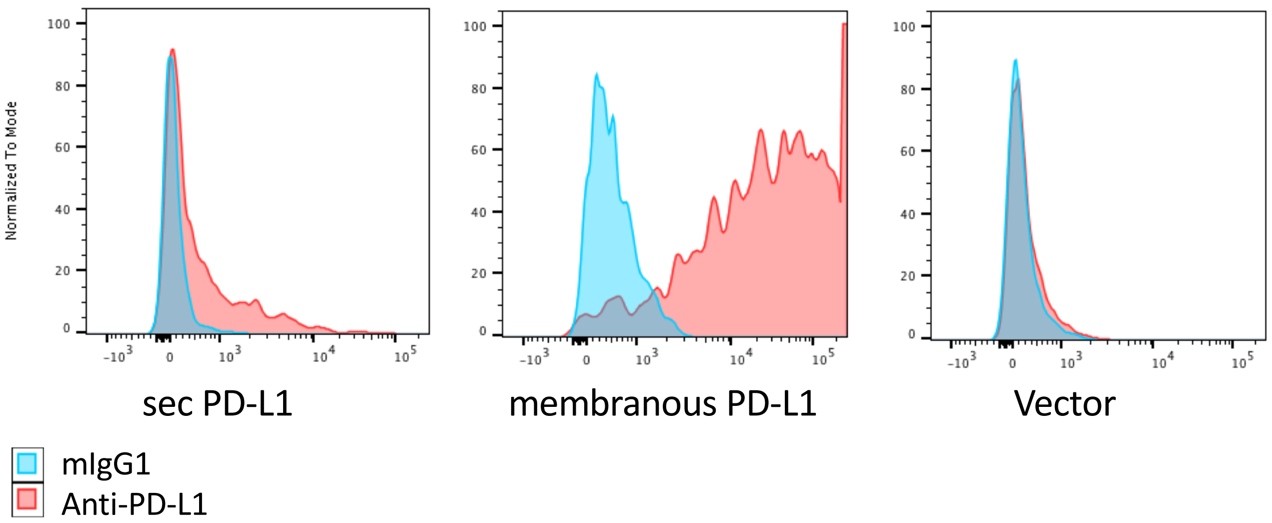

c.

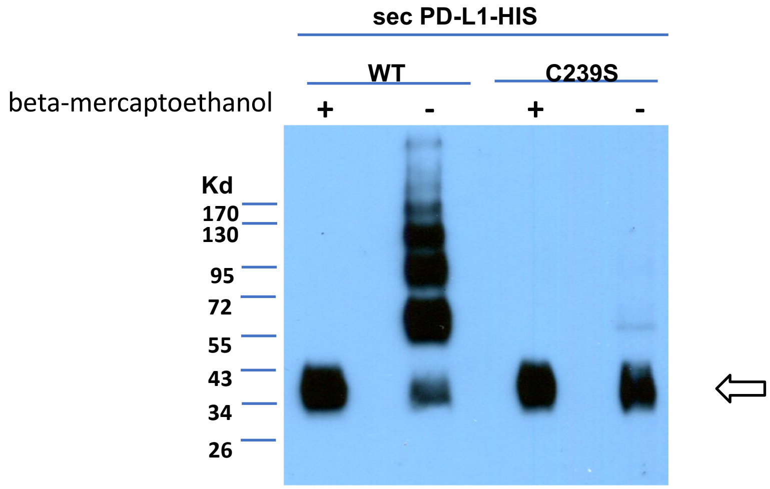

d. PCR schema

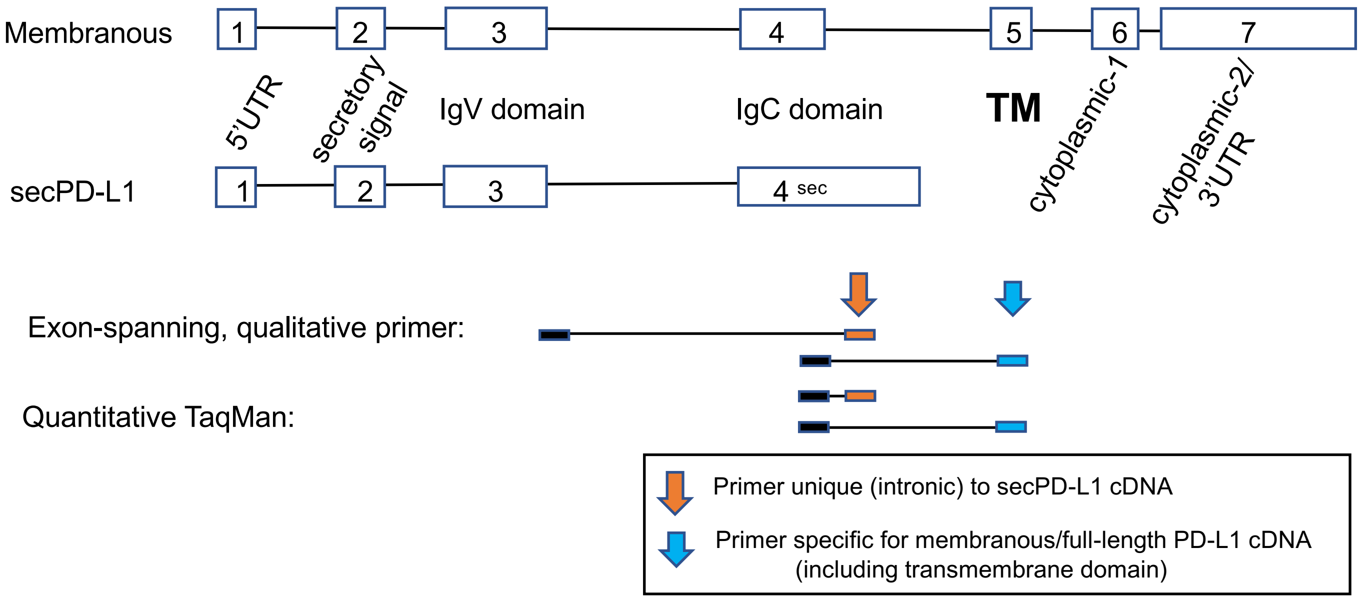

e. Exon-spanning, qualitative primer
PCR products (>300bp):

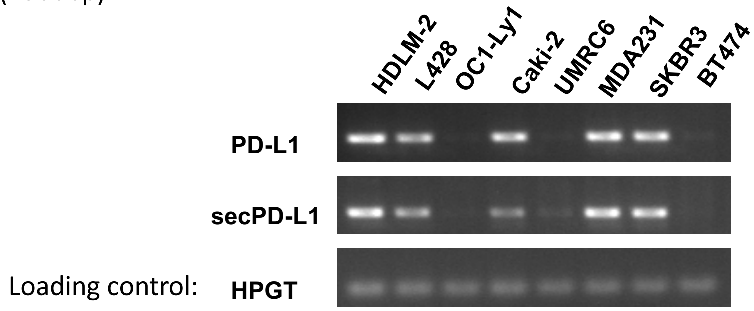

Supplemental Figure 1. (**a**) SecPD-L1 and full-length PD-L1 are distinguishable by qRT-PCR. SecPD-L1 and full-length PD-L1 share 227 amino acids but secPD-L1 has an 18 amino acid tail different from the full-length protein. (**b**) Flow cytometry of 293T transfected with vector, secPD-L1, or full-length PD-L1 (blue isotype control; red PD-L1 antibody (clone 339.6A2). (**c**) Western blot analysis of recombinant secPD-L1 and secPD-L1 with mutation of the cysteine 239 within the C-terminus, in reducing and non-reducing conditions (open arrow indicating monomeric PD-L1 and closed arrow indicating dimeric secPD-L1 protein). (**d**) Semiquantitative (exon-exon spanning) and (**e**) quantitative PCR schema to distinguish the 207 base pairs at the 3' end of the mRNA unique to the secPD-L1 transcript and 804 base pairs at the 3' end of full-length PD-L1 mRNA Full-length PD-L1 expression relative to secPD-L1 RNA expression by qRT-PCR of cancer cell lines in figure 2a.

Supplemental Figure 2.

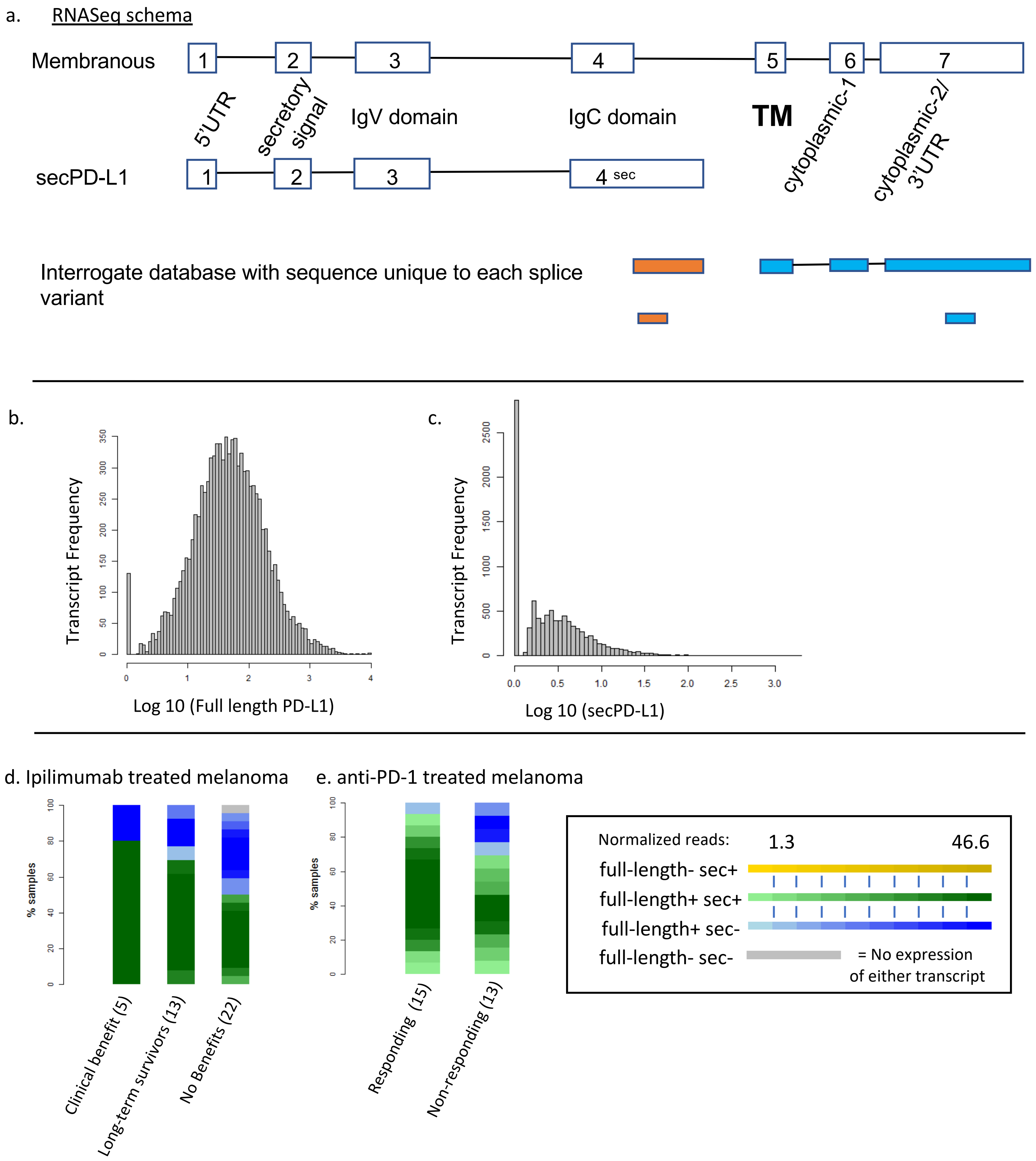

Supplemental Figure 2. SecPD-L1 and full-length PD-L1 expression are interrogatable by RNASeq analysis. (**a**) Transcriptomic strategy to distinguish the unique 3' end of the secPD-L1 from the 3' end of the full-length PD-L1 in RNASeq. (**b-c**) Frequency of tumors and amount of full-length PD-L1 or secPD-L1 expression in all tumors in TCGA. (**d-e**) Nearly all melanoma specimens from patients treated with CTLA-4 blockade or PD-1 blockade (nivolumab or pembrolizumab) expressed full-length PD-L1 and the majority express secPD-L1.

Supplemental Figure 3.

a.

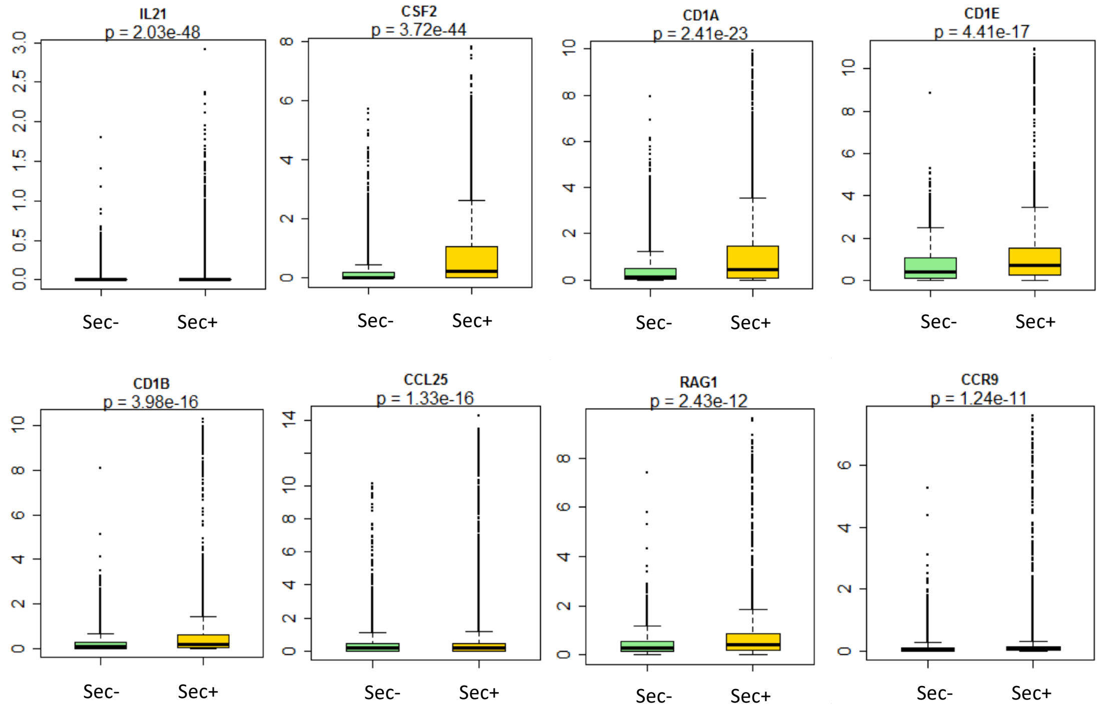

b.

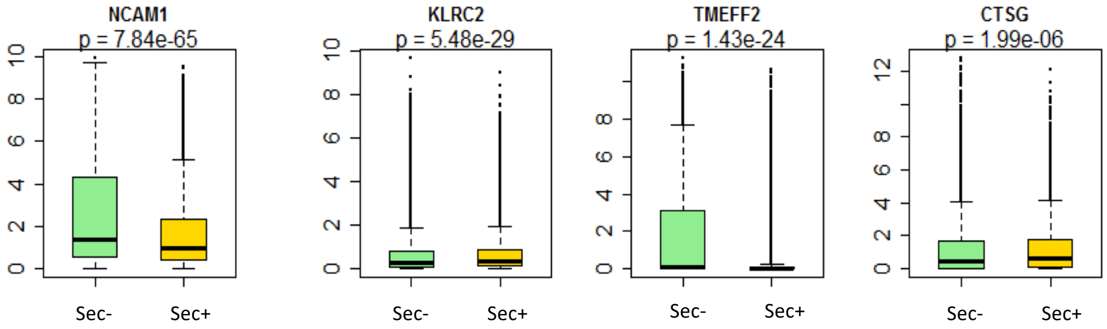

Supplemental Figure 3. Gene Set Enrichment Analysis (GEAS) shows differences in sec-expressing and no-sec-expressing cohorts of TCGA. (**a**) Within the 805 immune-related genes in the differential gene analysis, eleven transcripts are enriched in sec-expressing cohort: CD274/PD-L1, IFNγ and S100A8 (in Figure 4b), and IL21, CSF2, CD1A, CD1E, CD1B, CCL25, RAG1, CCR9; (**b**) while only four immune-related transcripts are enriched in no-sec-expressing cohort of TCGA: CTSG, KLRC2, NCAM1, TMEFF2.
